# Association between maternal fish consumption during pregnancy and preterm births: the Japan Environment and Children’s Study

**DOI:** 10.1265/ehpm.23-00084

**Published:** 2023-08-30

**Authors:** Kazue Ishitsuka, Mayumi Tsuji, Megumi Yamamoto, Rie Tanaka, Reiko Suga, Mami Kuwamura, Toshihide Sakuragi, Masayuki Shimono, Koichi Kusuhara, the Japan Environment and Children’s Study Group

**Affiliations:** 1 Department of Environmental Health, School of Medicine, University of Occupational and Environmental Health; 2 Departiment of Social Medicine, National Center for Child Health and Development, Tokyo, Japan; 3 Department of Environment and Public Health, National Institute for Minamata Disease, Minamata, Japan; 4 Regional Center for Japan Environment and Children’s Study, University of Occupational and Environmental Health, Kitakyushu, Japan; 5 Department of Pediatrics, University of Occupational and Environmental Health, Kitakyushu, Japan

**Keywords:** Fish, Birth cohort, Preterm births

## Abstract

**Background:**

Fish are a rich source of essential nutrients that protect against preterm birth. However, as fish can absorb environmental pollutants, their consumption can also increase the risk of preterm birth. This study aimed to assess whether maternal fish consumption during pregnancy is associated with preterm birth in a nationwide large Japanese cohort that consumed relatively high amounts and many types of fish.

**Methods:**

This study included 81,428 mother-child pairs enrolled in a nationwide prospective Japanese birth cohort study. Fish consumption was assessed using a validated food frequency questionnaire. Multivariate logistic regression was used to investigate the association of total consumption of fish, fatty fish and lean fish, fish paste, and seafood and clams with preterm birth, adjusted for potential confounders.

**Results:**

There was no association between overall fish consumption and preterm births. However, the highest quintile of fish paste consumption was significantly associated with an increased risk of preterm birth (odds ratio [OR]: 1.11; 95% confidence interval [CI: 1.04, 1.17]). The consumption of baked fish paste at least three times per week was significantly associated with preterm birth (OR: 1.20; 95% CI: 1.03, 1.40). Consumption of other types of fish, except fish paste, was not significantly associated with preterm birth risk.

**Conclusions:**

Fish paste consumption may increase the risk of preterm birth. Further studies are required to confirm this association.

**Supplementary information:**

The online version contains supplementary material available at https://doi.org/10.1265/ehpm.23-00084.

## Background

Preterm birth increases the risk of neurodevelopmental delays, developmental disorders, and respiratory and gastrointestinal impairments [1]. Maternal dietary intake during pregnancy is both a risk and protective factor for preterm births. Several epidemiological studies have shown that prenatal exposure to environmental pollutants, including methylmercury, persistent organic pollutants, and polychlorinated biphenyls, is associated with an increased risk of preterm birth [2]. Fish can absorb environmental pollutants, and thus, their consumption can also increase the risk of preterm birth. However, fish are also rich in n-3 fatty acids (n-3PUFA), proteins, and vitamin D, which are beneficial for the prevention of preterm birth [3, 4]. As such, the recommendation of fish consumption during pregnancy needs to consider a balanced diet between the favorable effects of beneficial nutrients and the unfavorable effects of toxic environmental pollutants [5–9].

However, epidemiological evidence on the association between fish consumption and preterm birth has been inconsistent. A pooled analysis from European birth cohort studies has shown that fish consumption during pregnancy is associated with a lower risk of preterm birth [10]. A study from China also showed that fish consumption reduces the risk of preterm birth [11]. In contrast, a linkage study of biomonitoring mercury in fish and birth records in the US found that high fish consumption was associated with an increased risk of preterm birth [12]. A prospective cohort study in the US also found that fish consumption increased the risk of preterm birth in overweight women, whereas fish consumption decreased the risk of preterm birth in underweight women [13]. Meanwhile, other epidemiological studies found no associations between fish consumption and preterm delivery [14, 15].

The Japanese have one of the highest fish consumption worldwide, and fish types consumed also vary, allowing the investigation of the association between fish consumption and birth outcomes. However, to our knowledge, no study has examined the association between fish consumption and birth outcomes in Japan. Thus, this study aimed to investigate the associations between each type of fish consumption and preterm births using data from a nationwide large birth cohort study.

## Materials and methods

### Study design and participants

**Fig. 1:**
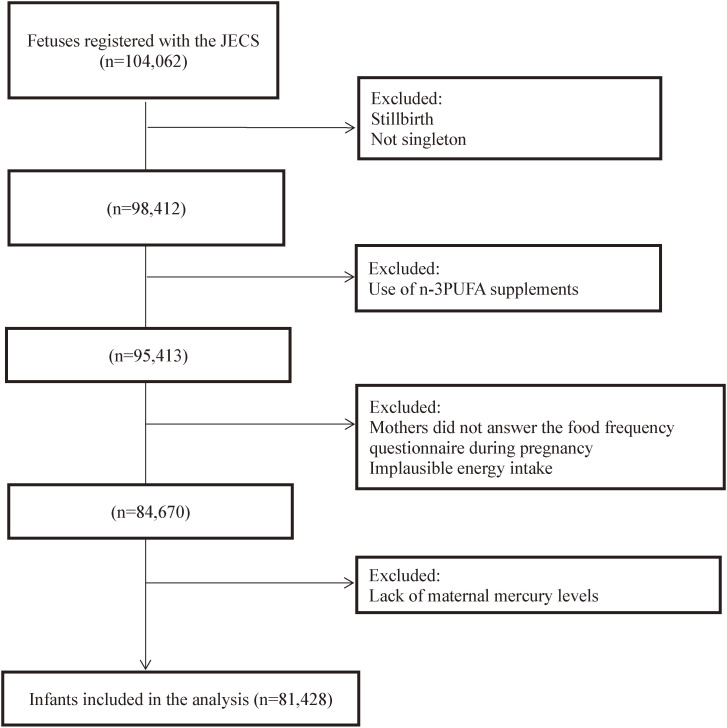
Flow diagram of participant selection.

The Japan Environment and Children’s Study (JECS) is an ongoing nationwide birth cohort study that has recruited expecting mothers at 15 Regional Centers distributed across a wide geographical area between January 2011 and March 2014. The design of the JECS has been previously described in detail [16–21]. Briefly, the main objective of the JECS was to investigate the influence of environmental chemical exposures on child health and development. Women were recruited throughout pregnancy (mean gestational age at recruitment: 13.2 weeks, standard deviation 8.4 weeks). Pregnant women were recruited at the clinic during prenatal checkups or at local government offices, where they were requested to register their pregnancy after learning about their conception, in regional centers located in urban and rural areas across Japan. The eligibility criteria were pregnant women who lived in the study area, and whose expected delivery date was between 2011 and 2014. A total of 104,062 fetal records were registered in the JECS (Fig. 1) [17]. This cohort study included 98,412 singleton and live births. Mothers who used n-3PUFA dietary supplements during pregnancy were excluded. Those who did not have information on maternal dietary intake or with excessively low (<4500 kJ) and high energy intake (≥20000 kJ) were also excluded to avoid misreporting in the food frequency questionnaire (FFQ) [22]. In total, 81,428 mother-infant pairs were included in the analysis.

### Outcomes

Gestational age and obstetric information were obtained using medical charts at delivery. Preterm birth was defined as birth a gestational age between 22 weeks and 36 weeks based on medical charts at delivery. Furthermore, we identified preterm births with the following two causes: induced preterm birth was defined as labor-induced or cesarean delivery, except for previous cesarean delivery, while spontaneous preterm birth was defined as vaginal birth without labor induction [23].

### Dietary intake

Dietary intake was assessed using the FFQ administered after pregnancy awareness. The questionnaire was administered during middle pregnancy (mean: 27.9 [standard deviation, 0.21] weeks of gestation). The FFQ is a semi-quantitative questionnaire that assesses dietary behaviors, major cooking methods, consumption frequency, and semi-quantitative portion sizes of foods and beverages [24]. The FFQ has been validated for use in large-scale Japanese epidemiological studies [24, 25]. Mothers answered their habitual frequency of consumption of 13 items of seafood and 14 items of processed fish from nine categories ranging from “less than once a month” to “seven times per day.” The type of fish consumed was categorized as follows: fatty fish (salmon, trout, sardines, tuna, bonito (katsuo), yellow tail, pacific saury, mackerel, eel), lean fish (cod, flatfish, sea bream), fish paste (baked fish paste (Chikuwa), steamed fish paste (Kamaboko), fried fish paste (Satsuma-age), seafood and clam (squid, octopus, shrimp, and clam), and others (fish egg, dried fish, and salted fish)) [15, 26].

The daily consumption of fish, energy, and nutrients was estimated using an ad hoc computer algorithm for the FFQ based on the Standard Tables of Food Composition in Japan 2010 [3]. The correlation coefficient between the FFQ and the 12-day weighted dietary records was 0.47 among Japanese women for the total fish consumption [24]. The amount of food and nutrient intake was energy adjusted using the density method [27].

### Covariates

Maternal sociodemographic characteristics and health-related behaviors were assessed through maternal questionnaires during pregnancy, including parity, marital status, household income, maternal education, employment status, physical activity, smoking, and alcohol consumption. Parity was categorized as primiparous or multiparous. Marital status was categorized as married and unmarried. Maternal education level was categorized into three groups based on the reported highest academic achievement: junior high school or high school (≤12 years), technical/vocational college or associated degree (13–15 years), and bachelor’s degree or higher (≥16 years). Employment status was categorized as either employed or unemployed. Smoking was categorized as follows: never smoking, quitting smoking, and current smoking. Alcohol consumption was categorized as follows: never consumed alcohol, ceased alcohol consumption, or continued to consume alcohol. Physical activity during pregnancy was assessed using the International Physical Activity Questionnaire [28–30]. Consumption of fruits, vegetables, and fermented foods (yogurt, cheese, miso soup, and fermented soybeans) was assessed using the FFQ [31].

Information on maternal height and weight was obtained from medical charts during the perinatal visits. Body mass index (BMI) was grouped into three categories: underweight (<18.5 kg/m^2^), normal weight (18.5–24.9 kg/m^2^), and overweight or obese (≥25 kg/m^2^) [32]. Data on pregnancy complications and infant congenital anomalies were obtained from medical charts at birth. Pregnancy complications included gestational diabetes and pregnancy-induced hypertension, and maternal uterus infection. Congenital anomalies included brain, sensory system, orofacial, limbs, congenital heart defects, abdominal anomalies, urogenital anomalies, neural tube defects, and chromosome abnormalities. Maternal total blood mercury levels during pregnancy were assessed using inductively coupled plasma-mass spectrometry with an Agilent 7700 inductively coupled plasma mass spectrometry (Agilent Technologies, Tokyo, Japan) [33]. Measurements of total blood mercury levels have been described previously [33].

### Statistical analyses

The frequency of consumption of each type of fish was analyzed. The amount of each type of fish and seafood consumed estimated by the density method was divided into quintiles. Maternal characteristics were then described according to quintiles of total maternal fish consumption during pregnancy. Multivariate logistic regression was performed to investigate the effects of fish consumption on preterm, spontaneous, and induced deliveries, adjusting for maternal age, parity, education, fetus complication, maternal complication, maternal physical activity, intake of energy, fruits, vegetables, fermented foods (yogurt, cheese, miso soup, fermented soybeans), pre-BMI, smoking status, and alcohol consumption during pregnancy. Two models were developed for the analysis: model 1 was adjusted for maternal age, parity, pre-BMI, smoking status, and alcohol consumption during pregnancy, and model 2 was adjusted for blood mercury and covariates used in model 1. Blood mercury levels were log-transformed prior to the regression analysis. We conducted complete case analyses. All statistical analyses were conducted using SAS version 9.4 (SAS Institute).

## Results

Preterm births occurred in 4.5% of births; of these, 1.9% and 2.4% were spontaneous and medically induced preterm births, respectively. Table 1 shows the maternal characteristics. Total fish consumption was higher in mothers who were older, multiparous, achieved the highest education level, had the highest BMI, and never smokers. The median (interquartile range) consumption of total fish, fatty fish, lean fish, fish paste, seafood and clams, and other seafoods during pregnancy was 28.7 (15.3, 46.4) g/day, 13.3 (6, 22.8) g/day, 0 (0, 2.8) g/day, 1.3 (0.4.0) g/day, 3.3 (0, 8.6) g/day, and 6.7 (2.3, 10.7) g/day, respectively. The group in the highest quintile of fish consumption was more likely to have higher protein, n-3PUFA, and Hg levels. The most frequently consumed type of fish paste was baked fish paste, followed by steamed and fried fish paste (Table S1).

**Table 1:** Characteristics of the mothers

	**Fish intake**									

	**Q1**		**Q2**		**Q3**		**Q4**		**Q5**	
	**n or mean**	**(%) or SD**	**n or mean**	**(%) or SD**	**n or mean**	**(%) or SD**	**n or mean**	**(%) or SD**	**n or mean**	**(%) or SD**
Age (years), mean (SD)	30.4	(5.1)	31.1	(5.0)	31.4	(4.9)	31.7	(4.8)	31.8	(4.9)
Parity, n (%)										
Primiparity	5735	(35.2)	5085	(31.2)	4779	(29.4)	4450	(27.3)	4346	(26.7)
Multiparity	10374	(63.7)	11058	(67.9)	11377	(69.9)	11705	(71.9)	11796	(72.4)
Missing	176	(1.1)	144	(0.9)	129	(0.8)	131	(0.8)	143	(0.9)
Education (years), n (%)										
≤12	6794	(41.7)	5887	(36.2)	5485	(33.7)	5240	(32.2)	5435	(33.4)
13–15	6641	(40.8)	6975	(42.8)	6998	(43.0)	7086	(43.5)	6844	(42.0)
≥16	2796	(17.2)	3370	(20.7)	3757	(23.1)	3919	(24.1)	3939	(24.2)
Missing	54	(0.3)	55	(0.3)	45	(0.3)	41	(0.3)	67	(0.4)
BMI before pregnancy (kg/m^2^), n (%)										
<18.5	2672	(16.4)	2661	(16.3)	2643	(16.2)	2548	(15.7)	2532	(15.6)
18.5–24.9	11864	(72.9)	11991	(73.6)	11998	(73.7)	12050	(74.0)	11871	(72.9)
≥25	1737	(10.7)	1630	(10.0)	1631	(10.0)	1681	(10.3)	1877	(11.5)
Missing	12	(0.1)	5	(0.0)	13	(0.1)	7	(0.0)	5	(0.0)
Smoking, n (%)										
Never smoked	8649	(53.1)	9388	(57.6)	9622	(59.1)	9802	(60.2)	9634	(59.2)
Stopped smoking	6553	(40.2)	6032	(37.0)	5922	(36.4)	5740	(35.2)	5854	(36.0)
Continued to smoke during pregnancy	930	(5.7)	740	(4.5)	633	(3.9)	637	(3.9)	672	(4.1)
Missing	153	(0.9)	127	(0.8)	108	(0.7)	107	(0.7)	125	(0.8)
Alcohol consumption, n (%)										
Non-alcohol users	8264	(50.8)	8139	(50.0)	8140	(50.0)	8117	(49.8)	8249	(50.7)
Stopped alcohol uses	7512	(46.1)	7587	(46.6)	7558	(46.4)	7600	(46.7)	7418	(45.6)
Alcohol users	424	(2.6)	447	(2.7)	489	(3.0)	476	(2.9)	515	(3.2)
Missing	85	(0.5)	114	(0.7)	98	(0.6)	93	(0.6)	103	(0.6)
Mercury (ng/g) (geometric mean, CV)*	3.01	(0.6)	3.37	(0.6)	3.64	(0.5)	3.96	(0.5)	4.44	(0.6)

CV, coefficient of variation; Q1, first quintile; Q2, second quintile; Q3, third quintile; Q4, fourth quintile; Q5, fifth quintile.

*Blood total mercury during pregnancy was examined.

Table 2 shows the multivariate logistic regression analysis on the association between amounts of energy-adjusted fish consumption and preterm during pregnancy. There were no associations between overall fish consumption and preterm birth. In contrast, the highest quintile of fish paste consumption was associated with a higher risk of preterm birth (odds ratio [OR]: 1.11; 95% confidence interval [CI: 1.04, 1.17]). Further adjustment of blood levels of mercury during pregnancy did not attenuate the association of fish paste intake with preterm birth (OR: 1.11; 95% CI: 1.04, 1.17). Except for consumption of fish paste, consumption of other types of fish was not significantly associated with preterm birth risk. The associations between fish consumption and preterm was not different between spontaneous and induced preterm births.

**Table 2:** Associations between maternal fish consumption and preterm birth during pregnancy

	**Preterm (<37 weeks)**				**Spontaneous preterm**				**Induced preterm**			
	**Model 1**		**Model 2**		**Model 1**		**Model 2**		**Model 1**		**Model 2**	
	**OR**	**(95%CI)**	**OR**	**(95%CI)**	**OR**	**(95%CI)**	**OR**	**(95%CI)**	**OR**	**(95%CI)**	**OR**	**(95%CI)**
Total fish												
Q1	Ref		Ref		Ref		Ref		Ref		Ref	
Q2	0.98	(0.92, 1.05)	0.97	(0.91, 1.04)	0.98	(0.89, 1.07)	0.97	(0.89, 1.07)	0.97	(0.88, 1.07)	0.97	(0.87, 1.07)
Q3	1.00	(0.94, 1.07)	0.99	(0.93, 1.06)	0.98	(0.89, 1.08)	0.97	(0.89, 1.07)	0.98	(0.89, 1.08)	0.97	(0.88, 1.07)
Q4	0.97	(0.91, 1.04)	0.96	(0.89, 1.02)	0.98	(0.89, 1.07)	0.97	(0.88, 1.06)	0.97	(0.88, 1.08)	0.96	(0.87, 1.07)
Q5	1.00	(0.93, 1.07)	0.98	(0.91, 1.05)	1.01	(0.92, 1.11)	1.00	(0.91, 1.10)	0.99	(0.90, 1.10)	0.98	(0.88, 1.08)
Fatty fish												
Q1	Ref		Ref		Ref		Ref		Ref		Ref	
Q2	0.97	(0.91, 1.03)	0.96	(0.90, 1.03)	0.93	(0.85, 1.02)	0.93	(0.84, 1.02)	0.94	(0.85, 1.04)	0.93	(0.84, 1.03)
Q3	1.00	(0.94, 1.07)	0.99	(0.93, 1.06)	1.02	(0.93, 1.12)	1.01	(0.92, 1.11)	1.04	(0.95, 1.15)	1.04	(0.94, 1.14)
Q4	0.97	(0.91, 1.04)	0.96	(0.90, 1.03)	0.99	(0.91, 1.09)	0.98	(0.90, 1.08)	0.99	(0.89, 1.09)	0.98	(0.88, 1.08)
Q5	0.97	(0.91, 1.04)	0.95	(0.89, 1.02)	0.95	(0.86, 1.04)	0.93	(0.85, 1.03)	0.95	(0.86, 1.05)	0.94	(0.85, 1.04)
Lean fish*												
No intake	Ref		Ref		Ref		Ref		Ref		Ref	
Intake	0.99	(0.95, 1.04)	0.98	(0.94, 1.03)	1.02	(0.96, 1.09)	1.02	(0.96, 1.09)	1.04	(0.97, 1.11)	1.03	(0.97, 1.11)
Fish paste												
Q1	Ref		Ref		Ref		Ref		Ref		Ref	
Q2	1.02	(0.88, 1.18)	1.02	(0.88, 1.18)	0.94	(0.77, 1.16)	0.94	(0.77, 1.16)	1.01	(0.81, 1.25)	1.01	(0.81, 1.25)
Q3	0.99	(0.94, 1.05)	0.99	(0.94, 1.05)	1.03	(0.95, 1.12)	1.03	(0.95, 1.12)	1.06	(0.97, 1.16)	1.06	(0.97, 1.16)
Q4	0.99	(0.94, 1.05)	0.99	(0.93, 1.05)	1.04	(0.96, 1.13)	1.04	(0.96, 1.13)	1.05	(0.96, 1.15)	1.05	(0.96, 1.15)
Q5	1.11	(1.04, 1.17)	1.10	(1.04, 1.17)	1.15	(1.06, 1.25)	1.15	(1.06, 1.25)	1.15	(1.06, 1.26)	1.15	(1.06, 1.26)
Seafood and clam												
Q1	Ref		Ref		Ref		Ref		Ref		Ref	
Q2	0.95	(0.88, 1.03)	0.95	(0.88, 1.03)	0.94	(0.85, 1.05)	0.94	(0.84, 1.05)	0.88	(0.79, 0.99)	0.88	(0.78, 0.99)
Q3	0.98	(0.93, 1.05)	0.98	(0.92, 1.04)	1.00	(0.92, 1.09)	0.99	(0.91, 1.08)	0.98	(0.90, 1.08)	0.98	(0.90, 1.07)
Q4	0.93	(0.87, 0.98)	0.92	(0.86, 0.98)	0.93	(0.86, 1.02)	0.93	(0.85, 1.01)	0.93	(0.85, 1.02)	0.93	(0.85, 1.02)
Q5	0.96	(0.91, 1.02)	0.95	(0.90, 1.01)	0.95	(0.87, 1.04)	0.94	(0.87, 1.03)	0.92	(0.84, 1.01)	0.91	(0.83, 1.00)
Other seafood												
Q1	Ref		Ref		Ref		Ref		Ref		Ref	
Q2	1.00	(0.93, 1.07)	0.99	(0.93, 1.06)	1.04	(0.94, 1.14)	1.03	(0.94, 1.13)	1.02	(0.92, 1.13)	1.01	(0.92, 1.12)
Q3	1.01	(0.95, 1.08)	1.01	(0.94, 1.08)	1.03	(0.94, 1.13)	1.03	(0.94, 1.13)	1.02	(0.92, 1.12)	1.01	(0.92, 1.12)
Q4	0.97	(0.90, 1.03)	0.96	(0.90, 1.02)	0.97	(0.88, 1.07)	0.96	(0.88, 1.06)	0.96	(0.87, 1.07)	0.96	(0.86, 1.06)
Q5	1.01	(0.94, 1.08)	1.00	(0.93, 1.06)	1.04	(0.94, 1.14)	1.03	(0.93, 1.13)	1.06	(0.96, 1.17)	1.05	(0.95, 1.16)

CI, confidence interval; ref, reference; Q1, first quintile; Q2, second quintile; Q3, third quintile; Q4, fourth quintile; Q5, fifth quintile.

*Lean fish consumption was not grouped to quintiles, quartiles, or tertiles because most of the women did not consume lean fish.

Model 1 was adjusted for maternal age, parity, education, fetus complication, maternal complication, maternal physical activity, intake of energy, fruits and vegetables, pre-BMI, smoking status, and alcohol consumption during pregnancy.

Model 2 was adjusted for covariates in Model 1 plus maternal blood mercury levels during pregnancy.

Table S2 shows the multivariate logistic regression analysis for the association between maternal frequency of fish consumption during pregnancy and preterm births. The highest frequency of baked fish paste consumption was associated with preterm birth (OR: 1.20; 95% CI: 1.03, 1.40). Further adjustment for blood levels of mercury did not attenuate the risk (OR: 1.19; 95% CI: 1.03, 1.38).

## Discussion

The association between fish consumption and birth outcomes in Japan has not been clarified to date. The current study found that a high previous consumption of fried fish paste, but not of other fish types, was significantly associated with an increased risk of preterm birth.

Large European cohort studies have shown that fatty fish consumption is associated with a decreased risk of preterm birth [34]. Fatty fish are rich in n3-PUFA, which can decrease the risk of preterm birth by improving placental function and increasing blood flow in the placenta [35]. Further, n-3PUFA protects from preterm birth through downregulation of prostacyclin [36, 37]. Prostacyclin has anti-aggregation and vasodilation effects, increases placental flow, and protects from preterm births [36, 37]. Furthermore, the anti-inflammatory properties of n-3PUFA could also be beneficial in preventing preterm births [38, 39]. Randomized controlled studies have shown that dietary supplement intake of n3-PUFA decreases the risk of preterm birth [4]. However, several epidemiological studies have failed to prove that n-3PUFA intake decreases preterm births [40]. Our results showed that fish consumption was not associated with a decreased risk of preterm births. This may be because environmental pollutants diminish protective effects of preterm, including methylmercury, persistent organic pollutants, and polychlorinated biphenyls [2].

The risk factors of preterm birth may differ in subtypes [41–44]. Epidemiological studies have shown that being underweight increases the risk of spontaneous preterm birth, but being overweight decreases the risk of spontaneous preterm birth [42–44]. In contrast, being overweight increases the risk of induced preterm birth [42–44]. This may be because overweight women have pregnancy complications related to induced delivery. In contrast, underweight women have higher risk for undernutrition, resulting in spontaneous preterm birth. Fish is rich in vitamin D and eicosapentaenoic acid, which can prevent spontaneous delivery [45–47]. Therefore, we analyzed the association between fish intake and each subtype of preterm birth (spontaneous and induced). However, our findings showed that fish consumption was not associated with spontaneous or induced preterm births.

Fish paste is widely and easily available at a low cost throughout East Asia, including in Japan. However, evidence on the association between fish paste consumption during pregnancy and birth outcomes is lacking. Our results showed that fish paste consumption was associated with an increased risk of preterm birth. Fish paste comprises a wide variety of fish types. Lean fish are commonly used for fish paste, but sometimes large fish, which may contain high levels of chemical pollutants, are also used. However, in this study, further adjustments for blood levels of mercury did not attenuate the association between fish paste intake and preterm birth. Our previous study showed that blood levels of mercury were not associated with preterm birth [48]. These findings suggest that mercury at current levels in Japan do not affect preterm birth. Another possible explanation for the effect of fish paste consumption on preterm births might be that the presence of food additives in fish paste influence preterm births. Further studies are needed to confirm the association between fish paste consumption and preterm births in children.

The strengths of the present study were its large sample size and the generalizability of the results to the Japanese population, which is characterized by high amounts and many types of fish consumption. Nevertheless, several limitations of this study also need to be considered. First, dietary intake was self-reported using the FFQ, introducing a possibility of misreporting [49, 50]. Second, despite careful adjustment for multiple confounders, unknown residual confounders might have existed because this was an observational study. Health consciousness could be a residual confounding factor. Although maternal education and healthy dietary patterns (e.g., consumption of fruits and vegetables) were added as covariates, residual confounders might have existed.

## Conclusion

Consumption of fatty fish and lean fish was not associated with preterm birth, but a high consumption of fish paste increases the risk of preterm birth. Future studies are needed to confirm these associations in other populations and reveal the underlying mechanisms.

## Abbreviations

BMI: body mass index
FFQ: food frequency questionnaire
JECS: Japan Environment and Children’s Study

## Supplementary information

**Additional file 1:** **Table S1.** Frequency of fish and seafood intake during pregnancy. **Table S2.** Results of logistic regression analysis (frequency of fish intake and preterm during pregnancy).

## Declarations

### Availability of data and material

Data are unsuitable for public deposition due to ethical restrictions and the legal framework in Japan. It is prohibited by the Act on the Protection of Personal Information (Act No. 57 of 30 May 2003, amendment on 9 September 2015) to publicly deposit data containing personal information. Ethical Guidelines for Medical and Health Research Involving Human Subjects enforced by the Japan Ministry of Education, Culture, Sports, Science and Technology and the Ministry of Health, Labour and Welfare also restrict the open sharing of epidemiologic data. All inquiries about access to data should be sent to jecs-en@nies.go.jp. The person responsible for handling enquiries sent to this e-mail address is Dr Shoji F. Nakayama, JECS Programme Office, National Institute for Environmental Studies.
