# Supplementary material for: Association between maternal fish consumption during pregnancy and preterm births: the Japan Environment and Children’s Study
Source: Environ Health Prev Med. 2023 Aug 30;28:47. doi: 10.1265/ehpm.23-00084 (PMC10480610; doi:10.1265/ehpm.23-00084)
Supplement: Supplementary file 1 — Additional file 1: Table S1. Frequency of fish and seafood intake during pregnancy. Table S2. Results of logistic regression analysis (frequency of fish intake and preterm during pregnancy). [file ehpm-28-047-s001.docx]

# Table S1. Frequency of fish and seafood intake during pregnancy

|  | Frequency of intake | | | | | | | |
| --- | --- | --- | --- | --- | --- | --- | --- | --- |
|  | Less than 1/month | | 1-3/month | | 1-2/week | | 3 and more/week | |
|  | n | (%) * | n | (%)* | n | (%)* | n | (%)* |
| Fatty fish |  |  |  |  |  |  |  |  |
| Tuna | 37424 | (46.0) | 33469 | (41.1) | 9717 | (11.9) | 818 | (46.0) |
| Salmon | 51764 | (63.6) | 25657 | (31.5) | 3723 | (4.6) | 284 | (63.6) |
| Pacific saury/mackerel | 52635 | (64.6) | 25782 | (31.7) | 2869 | (3.5) | 142 | (64.6) |
| Horse mackerel/sardine | 33828 | (41.5) | 40235 | (49.4) | 6949 | (8.5) | 416 | (41.5) |
| Yellow tail | 45698 | (56.1) | 30221 | (37.1) | 5102 | (6.3) | 407 | (56.1) |
| Lean fish |  |  |  |  |  |  |  |  |
| Cod/flatfish | 69522 | (85.4) | 10971 | (13.5) | 874 | (1.1) | 61 | (85.4) |
| Sea bream | 59341 | (72.9) | 20007 | (24.6) | 1976 | (2.4) | 104 | (72.9) |
| Fish paste |  |  |  |  |  |  |  |  |
| Baked fish paste | 37622 | (46.2) | 31686 | (38.9) | 10526 | (12.9) | 1594 | (46.2) |
| Steamed fish paste | 49916 | (61.3) | 24720 | (30.4) | 5969 | (7.3) | 823 | (61.3) |
| Fried fish paste | 52606 | (64.6) | 23502 | (28.9) | 4768 | (5.9) | 552 | (64.6) |
| Seafood |  |  |  |  |  |  |  |  |
| Shrimp | 34951 | (42.9) | 39500 | (48.5) | 6430 | (7.9) | 547 | (42.9) |
| Clam | 49160 | (60.4) | 27672 | (34.0) | 4242 | (5.2) | 354 | (60.4) |
| Squid | 52401 | (64.4) | 26259 | (32.2) | 2595 | (3.2) | 173 | (64.4) |
| Octopus | 59138 | (72.6) | 20707 | (25.4) | 1501 | (1.8) | 82 | (72.6) |

*The percentages were the column percentages.

# Table S2. Results of logistic regression analysis (frequency of fish intake and preterm during pregnancy)

|  | Preterm | | | | | |
| --- | --- | --- | --- | --- | --- | --- |
|  | Model 1 | | | Model 2 | | |
|  | OR | (95%CI) | | OR | (95%CI) | |
| Salmon |  |  |  |  |  |  |
| Less than 1/month | Ref |  |  | Ref |  |  |
| 1-3/month | 0.97 | (0.93, | 1.02) | 0.97 | (0.93, | 1.02) |
| 1-2/week | 1.00 | (0.94, | 1.08) | 1.00 | (0.93, | 1.07) |
| 3 and more/week | 1.27 | (1.04, | 1.56) | 1.31 | (1.08, | 1.59) |
| Horse mackerel/sardine |  |  |  |  |  |  |
| Less than 1/month | Ref |  |  | Ref |  |  |
| 1-3/month | 0.93 | (0.89, | 0.98) | 0.93 | (0.88, | 0.97) |
| 1-2/week | 1.00 | (0.90, | 1.10) | 0.98 | (0.89, | 1.09) |
| 3 and more/week | 1.23 | (0.87, | 1.72) | 1.14 | (0.82, | 1.58) |
| Pacific saury/mackerel |  |  |  |  |  |  |
| Less than 1/month | Ref |  |  | Ref |  |  |
| 1-3/month | 0.99 | (0.94, | 1.03) | 0.98 | (0.94, | 1.03) |
| 1-2/week | 0.98 | (0.90, | 1.06) | 0.97 | (0.90, | 1.05) |
| 3 and more/week | 0.99 | (0.74, | 1.33) | 1.01 | (0.76, | 1.34) |
| Yellowtail |  |  |  |  |  |  |
| Less than 1/month | Ref |  |  | Ref |  |  |
| 1-3/month | 0.99 | (0.94, | 1.03) | 0.98 | (0.93, | 1.02) |
| 1/week and more than | 0.91 | (0.81, | 1.03) | 0.90 | (0.80, | 1.01) |
| 3 and more/week | 1.88 | (1.23, | 2.89) | 1.69 | (1.11, | 2.58) |
| Tuna |  |  |  |  |  |  |
| Less than 1/month | Ref |  |  | Ref |  |  |
| 1-3/month | 0.96 | (0.92, | 1.01) | 0.95 | (0.91, | 0.99) |
| 1/week and more than | 1.05 | (0.96, | 1.15) | 1.02 | (0.94, | 1.12) |
| 3 and more/week | 1.06 | (0.78, | 1.44) | 1.01 | (0.75, | 1.35) |
| Sea bream |  |  |  |  |  |  |
| Less than 1/month | Ref |  |  | Ref |  |  |
| 1-3/month | 0.94 | (0.88, | 1.00) | 0.93 | (0.87, | 0.99) |
| 1-2/week | 1.05 | (0.86, | 1.28) | 1.03 | (0.85, | 1.26) |
| 3 and more/week | 1.68 | (0.87, | 3.26) | 1.45 | (0.76, | 2.79) |
| Cod/flatfish |  |  |  |  |  |  |
| Less than 1/month | Ref |  |  | Ref |  |  |
| 1-3/month | 1.00 | (0.95, | 1.05) | 0.99 | (0.95, | 1.04) |
| 1-2/week | 1.12 | (0.99, | 1.28) | 1.12 | (0.98, | 1.27) |
| 3 and more/week | 1.61 | (0.96, | 2.70) | 1.56 | (0.95, | 2.58) |
| Baked fish paste |  |  |  |  |  |  |
| Less than 1/month | Ref |  |  | Ref |  |  |
| 1-3/month | 1.00 | (0.95, | 1.05) | 1.00 | (0.95, | 1.04) |
| 1-2/week | 1.12 | (1.05, | 1.20) | 1.12 | (1.05, | 1.19) |
| 3 and more/week | 1.20 | (1.03, | 1.40) | 1.19 | (1.03, | 1.38) |
| Steamed fish paste |  |  |  |  |  |  |
| Less than 1/month | Ref |  |  | Ref |  |  |
| 1-3/month | 1.01 | (0.96, | 1.06) | 1.01 | (0.96, | 1.06) |
| 1-2/week | 1.11 | (1.02, | 1.20) | 1.11 | (1.02, | 1.20) |
| 3 and more/week | 0.97 | (0.78, | 1.22) | 0.92 | (0.74, | 1.14) |
| Fried fish paste |  |  |  |  |  |  |
| Less than 1/month | Ref |  |  | Ref |  |  |
| 1-3/month | 1.01 | (0.96, | 1.06) | 1.01 | (0.96, | 1.06) |
| 1-2/week | 1.12 | (1.03, | 1.22) | 1.12 | (1.02, | 1.22) |
| 3 and more/week | 1.12 | (0.87, | 1.44) | 1.12 | (0.88, | 1.43) |
| Shrimp |  |  |  |  |  |  |
| Less than 1/month | Ref |  |  | Ref |  |  |
| 1-3/month | 0.97 | (0.93, | 1.01) | 0.96 | (0.92, | 1.01) |
| 1-2/week | 0.97 | (0.89, | 1.05) | 0.96 | (0.88, | 1.04) |
| 3 and more/week | 1.00 | (0.77, | 1.30) | 1.03 | (0.80, | 1.32) |
| Clam |  | , |  |  |  |  |
| Less than 1/month | Ref |  |  | Ref |  |  |
| 1-3/month | 0.98 | (0.94, | 1.02) | 0.97 | (0.93, | 1.02) |
| 1-2/week | 1.07 | (0.98, | 1.18) | 1.06 | (0.97, | 1.17) |
| 3 and more/week | 0.84 | (0.59, | 1.21) | 0.96 | (0.70, | 1.32) |
| Squid |  |  |  |  |  |  |
| Less than 1/month | Ref |  |  | Ref |  |  |
| 1-3/month | 0.99 | (0.95, | 1.04) | 0.99 | (0.94, | 1.03) |
| 1-2/week | 1.07 | (0.95, | 1.20) | 1.06 | (0.94, | 1.19) |
| 3 and more/week | 0.77 | (0.47, | 1.28) | 0.80 | (0.50, | 1.28) |
| Octopus |  |  |  |  |  |  |
| Less than 1/month | Ref |  |  | Ref |  |  |
| 1-3/month | 0.98 | (0.93, | 1.02) | 0.97 | (0.92, | 1.02) |
| 1-2/week | 1.08 | (0.92, | 1.25) | 1.07 | (0.91, | 1.24) |
| 3 and more/week | 0.39 | (0.14, | 1.07) | 0.44 | (0.18, | 1.10) |

OR, odds ratio; ref, reference

Model 1 was adjusted for maternal age, parity, education, fetus complication, maternal complication, maternal physical activity, intake of energy, fruits and vegetables, pre-BMI, smoking status, and alcohol consumption during pregnancy.

Model 2 was adjusted for covariates in Model 1 and maternal blood levels of mercury during pregnancy.
